# The research perspectives and frontiers on radiotherapy for hepatocellular carcinoma: a bibliometric analysis and systematic review

**DOI:** 10.3389/fonc.2025.1601207

**Published:** 2025-12-16

**Authors:** Tao He, Hong Xiao, Bin Yi, Chengyan Zhou, Juan Yang, Ke Sun, Jieyu Zou

**Affiliations:** 1 Department of Hepatobiliary Surgery, Chengdu Second People’s Hospital, Chengdu, Sichuan, China; 2 Department of Oncology, Chengdu Second People’s Hospital, Chengdu, Sichuan, China

**Keywords:** radiotherapy, hepatocellular carcinoma, transarterial chemoembolization, portal vein tumor thrombosis, sorafenib, bibliometric analysis, systematic review

## Abstract

**Introduction:**

Research into radiotherapy(RT) for hepatocellular carcinoma(HCC) has significantly advanced, underscoring its critical role in systemic therapy. However, there remains a notable lack of quantitative and analytical studies specifically addressing RT in the context of HCC. This study aims to evaluate the evolution of RT research and provide a comprehensive analysis of RT studies using bibliometric citation analysis.

**Methods:**

A literature search was conducted on July 2, 2024, to extract relevant publications from the Science Citation Index-Expanded(SCIE) of the Web of Science Core Collection(WoSCC), spanning the years 2014 to 2023. Bibliometric and quantitative assessments of the collected publications were performed using visualization tools such as CiteSpace and VOSviewer.

**Results:**

The research identified 822 manuscripts authored by 4681 researchers from 1083 institutions across 17 countries, published in 225 journals, and involving 16183 co-cited references from 2282 journals. The United States led in publication volume with 147 articles (17.9%), showing close collaboration with Canada and China. Key areas of focus included the use of RT for HCC with portal vein tumor thrombosis and its combination with transarterial chemoembolization for advanced HCC treatment, which have garnered significant global interest. Recently, particle therapy and the integration of targeted therapies and immunotherapy with RT have emerged as critical themes and frontiers in this research domain.

**Discussion:**

This study provides a comprehensive analysis and summary of the current research trends and emerging insights regarding RT in HCC. RT combined with transarterial chemoembolization(TACE) for portal vein tumor thrombosis(PVTT) in HCC has received widespread attention, while particle therapy and combination targeted immunotherapies with RT are essential topics and emerging areas of interest. The resultant synthesis offers a substantive foundation for healthcare practitioners and researchers, providing pivotal meaningful insights that can guide and inspire further research efforts in this field.

## Graphical Abstract

**Figure:**
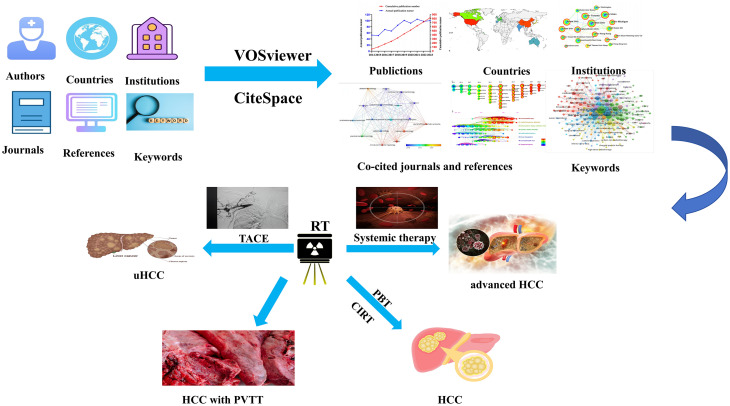
The research perspectives and frontiers on radiotherapy for hepatocellular carcinoma from 2014 to 2023.

## Introduction

Primary liver cancer(PLC), the sixth most prevalent malignancy worldwide, accounted for approximately 906,000 new cases and 830,000 deaths in 2020, representing the third leading cause of cancer-related mortality globally (1). Notably, PLC ranked as the second most lethal malignancy across both genders from 2005 to 2020 and emerged as the primary cause of cancer death in individuals aged 20–59 years during this period (2). Hepatocellular carcinoma(HCC), constituting 75%-86% of PLC cases, remains the dominant histological subtype (1). Despite advances in multimodal therapeutic approaches including surgical resection and liver transplantation(LT), HCC management continues to face significant challenges: five-year recurrence rates reach 70% post-surgical resection/ablation and 35% following LT (3–6). The scarcity of donor organs and procedure-related morbidity further emphasize the imperative for optimized treatment strategies, necessitating an enhanced understanding of HCC pathogenesis and therapeutic optimization.

Historically characterized as radioresistant due to cirrhotic liver tissue’s reduced radiation tolerance, HCC management through conventional two-dimensional radiotherapy was constrained by radiation-induced liver disease(RILD) risks and inadequate dose conformality, limiting its application to palliative care in advanced disease (7). The advent of precision radiotherapy modalities-particularly stereotactic body radiotherapy(SBRT), intensity-modulated radiotherapy(IMRT), and image-guided radiotherapy(IGRT)-has transformed HCC management through millimeter-level targeting accuracy. These advancements enable tumoricidal dose delivery while sparing functional hepatic parenchyma, establishing radiotherapy as a definitive treatment modality (8, 9). This paradigm shift is reflected in evolving global guidelines: while the 2014 American Association for the Study of Liver Diseases(AASLD) guidelines restricted radiotherapy to portal vein tumor thrombosis(PVTT) palliation, the 2023 update recommends radiotherapy as first-line therapy for Barcelona Clinic Liver Cancer(BCLC) stage A patients ineligible for resection, including those with lesions >3cm (10, 11). The National Comprehensive Cancer Network(NCCN) guidelines similarly expanded radiotherapy indications - recognizing SBRT as definitive therapy for ≤5 cm lesions in 2018, then incorporating radiotherapy as neoadjuvant therapy for LT candidates and adjuvant treatment for microscopic residual disease (12, 13). Current European Association for the Study of the Liver(EASL) guidelines position SBRT as first-line therapy for early-stage HCC and a component of combination therapy for intermediate-stage disease (14). Selective internal radiotherapy (SIRT) demonstrates particular utility as a bridging strategy for LT candidates with contraindications to transarterial chemoembolization(TACE) or systemic therapy, per the European Society for Medical Oncology(ESMO) recommendations (15). Emerging modalities including proton beam therapy(PBT) and carbon ion radiotherapy(CIRT) exploit unique physical properties to optimize outcomes in large HCC lesions (16, 17). Modern radiotherapy synergizes effectively with systemic therapies, as evidenced by superior response and overall survival(OS) TACE-RT combination therapy versus TACE alone (18, 19). A recent study declared that local RT after sorafenib significantly prolonged OS compared to sorafenib alone in BCLC stage C HCC, particularly for patients treated with sorafenib for over 12 weeks (23.6 vs. 15.3 months) (20). These clinical advances underscore radiotherapy’s evolution from palliative intervention to the cornerstone of curative-intent multimodal strategies.

Bibliometric analysis quantitatively evaluates research trends and academic impact through statistical analysis and data visualization, providing critical insights into field development (21). The insights gained from these analyses, especially through visual representations, offer new perspectives on HCC research (22–24). While existing HCC bibliometric studies have identified emerging directions in immunotherapy (25, 26), targeted therapy (27), and TACE (28), no comprehensive analysis currently exists for radiotherapy applications in HCC. Our study addresses this gap through multidimensional quantitative evaluation, aiming to elucidate RT’s evolving role in HCC management and inform future research directions.

## Materials and methods

### Database selection and search strategy

In this study, we employed bibliometric analysis to comprehensively examine the literature on RT in HCC. Our research utilized the Web of Science Core Collection(WoSCC), a robust search platform encompassing primary citation sources and publishing peer-reviewed journals and conference proceedings. On July 2, 2024, we retrieved literature published between 2014 and 2023 from the Science Citation Index-Expanded(SCIE) within WoSCC. Our primary search terms included “primary hepatic cancer,” “hepatocellular carcinoma,” “primary liver cancer,” “primary liver carcinoma,” AND “stereotactic body radiation therapy,” “three-dimensional conformal radiation therapy,” “proton beam radiation therapy,” “intensity-modulated radiation therapy,” “external beam radiation therapy”,”radiotherapy.” The detailed search strategy is presented in **Supplementary File S1**.

### Inclusion criteria

Inclusion criteria were restricted to English-language, peer-reviewed articles and reviews to ensure data relevance and quality.

### Data extraction and deduplication process

We exported the literature records using the “Full record and cited references” option, formatting the output in plain text. The texts included: (1) General information: Author,s the affiliated countries and organizations, year of publications, and journals. (2) Study design, references, and keywords. Two independent reviewers screened the raw data and excluded duplicates and irrelevant documents.

### Quality assessment

**Figure 1:**
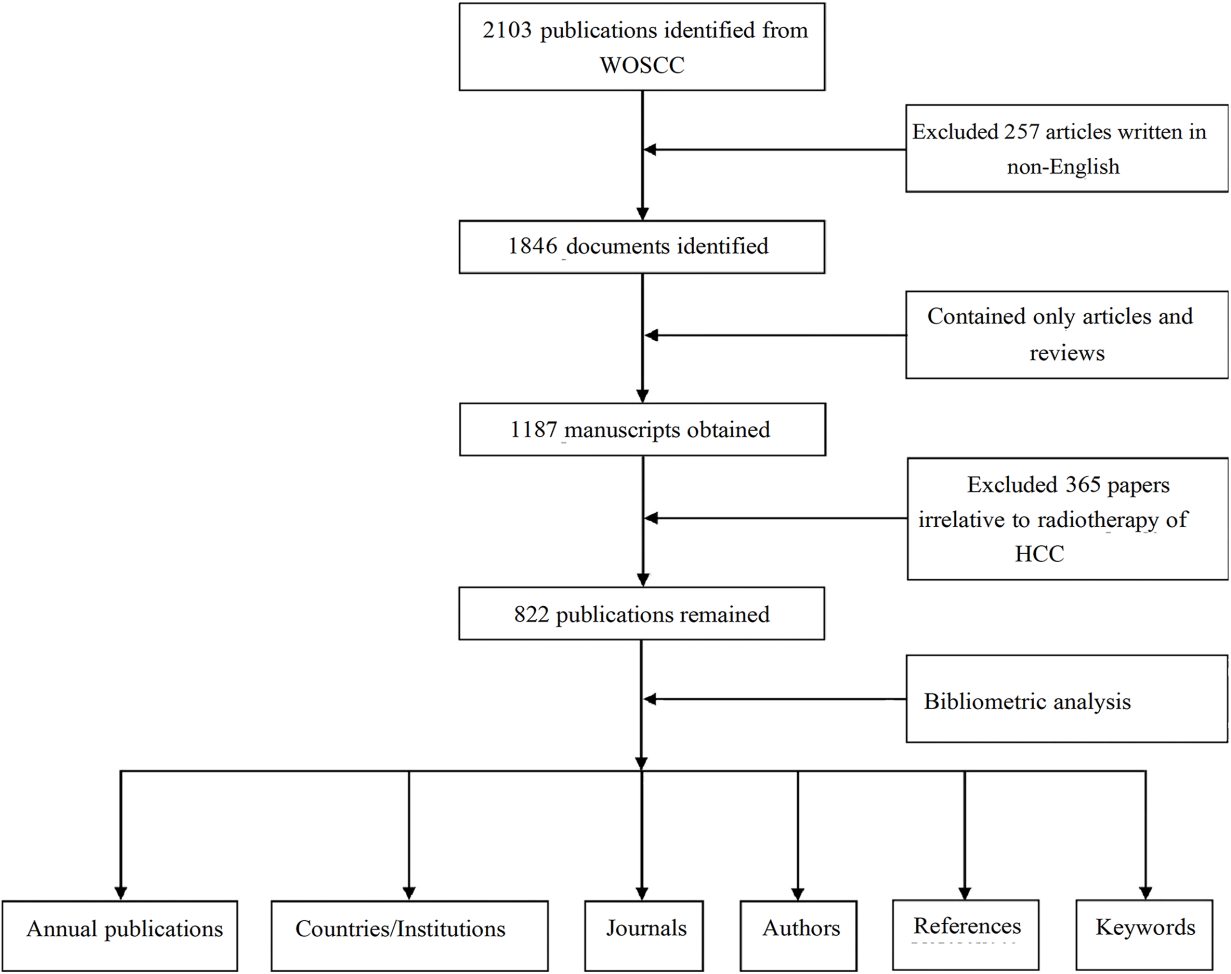
A flowchart of the research process to retrieve documents on RT and HCC from the WosCC database.

To ensure consistency, two reviewers independently piloted data extraction on random documents. Results were discussed to align the criteria. During extraction, two researchers independently collected data, discussing discrepancies, and a third researcher resolved any unresolved differences. Our study adheres to the Consolidated Criteria for Reporting Qualitative Research (COREQ) (29). The study flowchart is presented in **Figure 1**.

### Statistical analysis and visualization

The annual publication counts were tabulated and analyzed using Excel 2019, with trends in HCC radiotherapy publications graphically represented using GraphPad Prism (version 8.0.2). We employed CiteSpace (version 6.2 R6) to evaluate institutional affiliations, co-cited references, dual map overlays, and emerging keyword trends, using a time-slice setting of one year, a top N of 50, and a clustering resolution parameter k of 15 from 2014 to 2023. Pruning: network pruning method. Modularity Q(Q-value): Clustering module value, it is generally assumed that Q>0.3 means that the clustering structure is significant. Weighted mean silhouette S(S-value): The average silhouette value of the cluster, it is generally considered that the S>0.5 clustering class is reasonable, and S>0.7 means that the clustering is convincing. In the visual network maps, nodes in the visual network maps were color-coded to reflect chronology, with warmer colors indicating more recent contributions. Analyses of authors, co-cited authors, journals, co-cited journals, and keywords were conducted using VOSviewer(version 1.6.18). In VOSviewer, set ‘Attraction’ to 2 and ‘Repulsion’ to -1 in the layout settings. Then, set ‘Resolution’ to 2 in the clustering settings. In the visualization, the ‘weights’ on the scale are normalized by ‘documents’, while the ‘size variation’ in labels is displayed as ‘circles’, with a maximum value of 30. Collaborative networks were visualized using SCImago Graphics Beta(version 1.0.18). Furthermore, the visual knowledge graph consisted of nodes and linear connections. Each node in the graph represented a key point, and the node’s size represented the frequency of occurrence and citation.

## Results

This study analyzed 822 manuscripts authored by 4,681 researchers from 1,083 institutions across 17 countries. These included 800 articles and 22 reviews, published in 225 journals. The manuscripts cited 16,183 references from 2,282 distinct journals.

### Trends in publications

**Figure 2:**
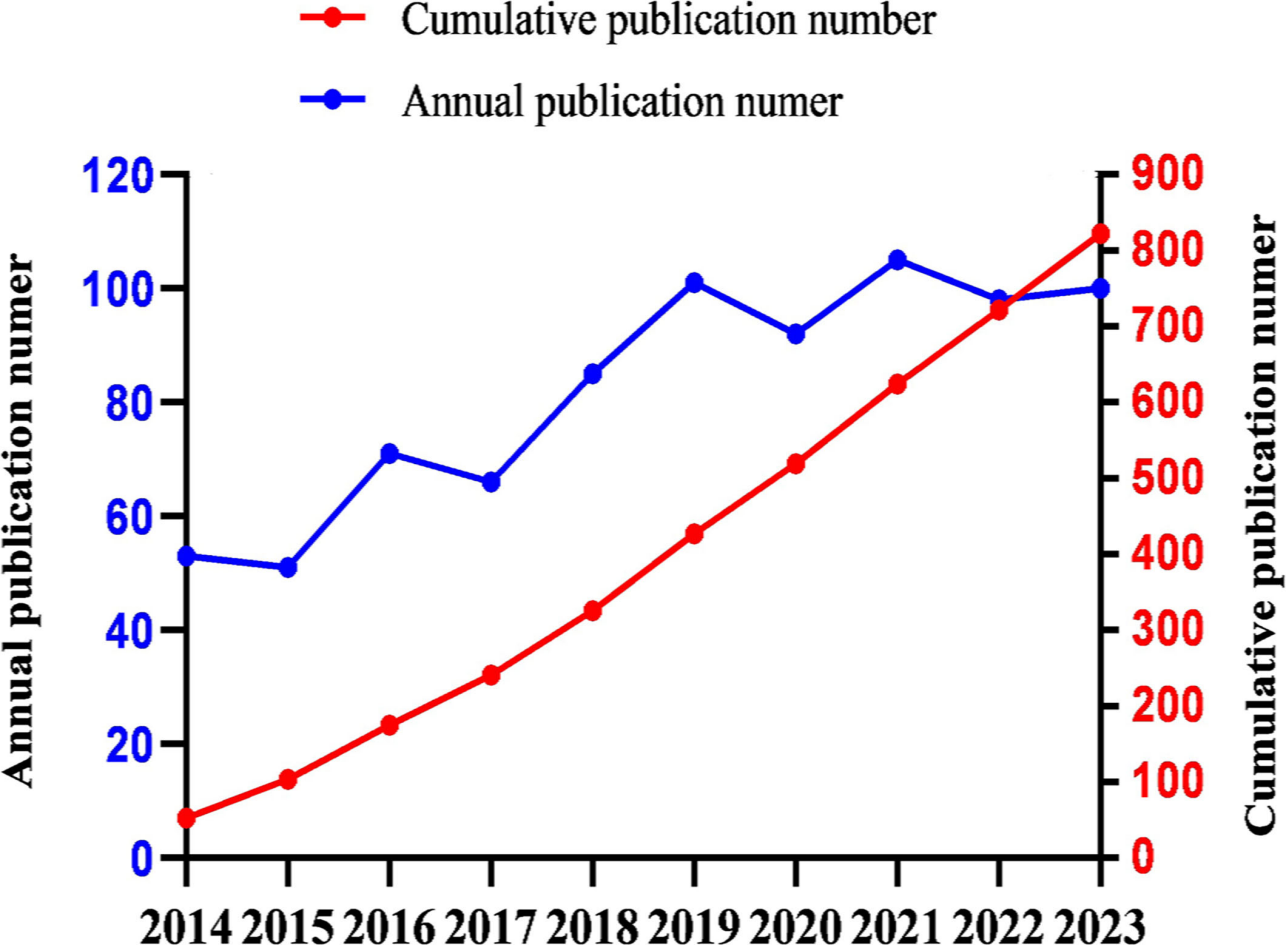
Number of publications by year from 2014 to 2023.

The annual publication output can provide insights into the evolving trends within the field. Over the past decade, the number of publications related to RT and HCC has exhibited a steady upward trajectory (**Figure 2**). The predictive equation(linear simulation trend) for the cumulative publication count(Y) by the year of publication(x) is given by Y = 87.436x-176089, with an R-squared value of 99% (R²=0.9914), indicating a reliable forecast. On average, the annual output over the last ten years is 82.2 publications, with 30% of these years exceeding 100 publications(included 2019, 2021 and 2023). Notably, 2021 recorded the highest number of publications (n=105).

### Geographic and institutional distribution

**Figure 3:**
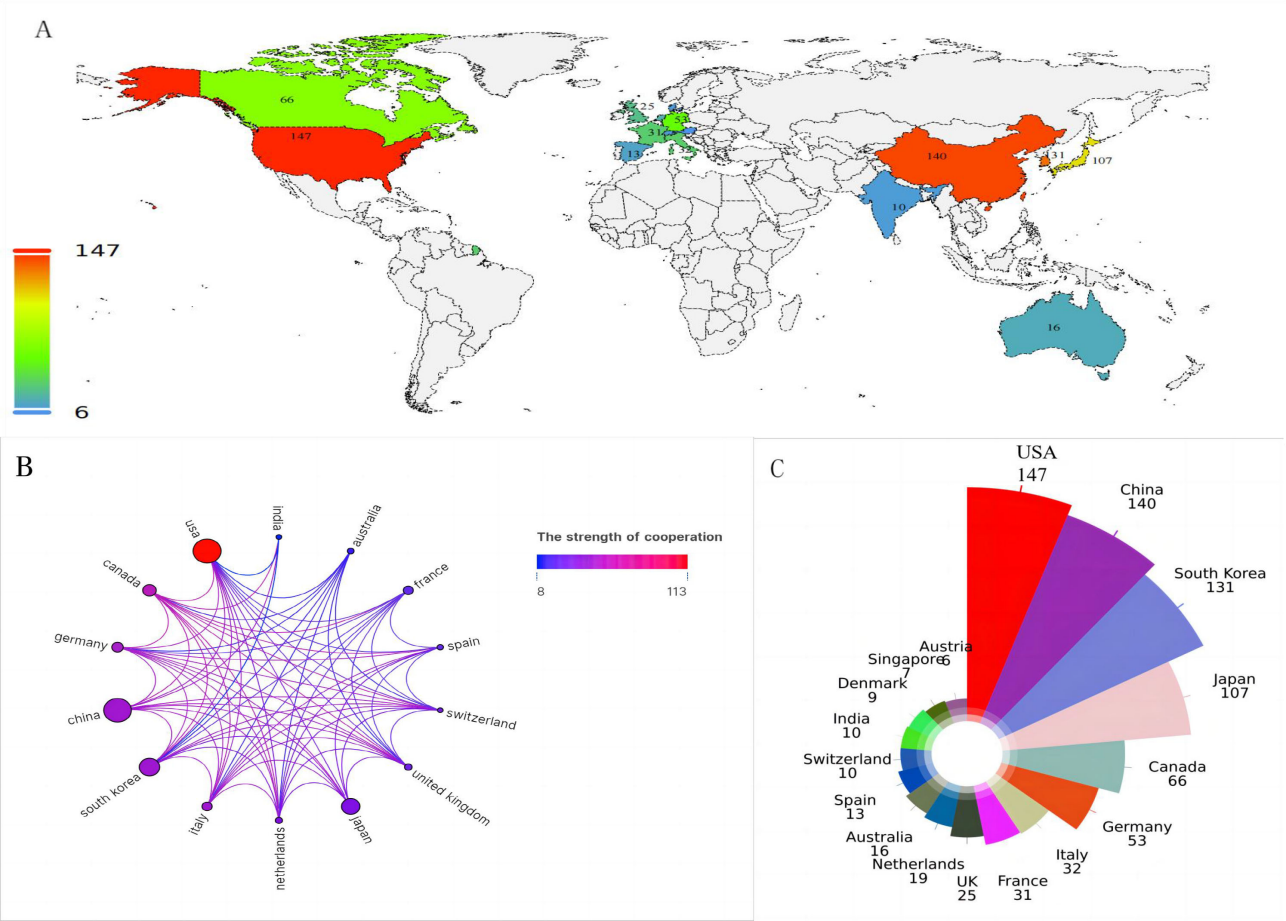
**(A)** The national geographic mapping of publications. **(B)** The network map of countries cooperation (the size of the node represents the number of publications, and the color of the line shows the strength of cooperation). **(C)** All countries in terms of number of publications.

**Figure 4:**
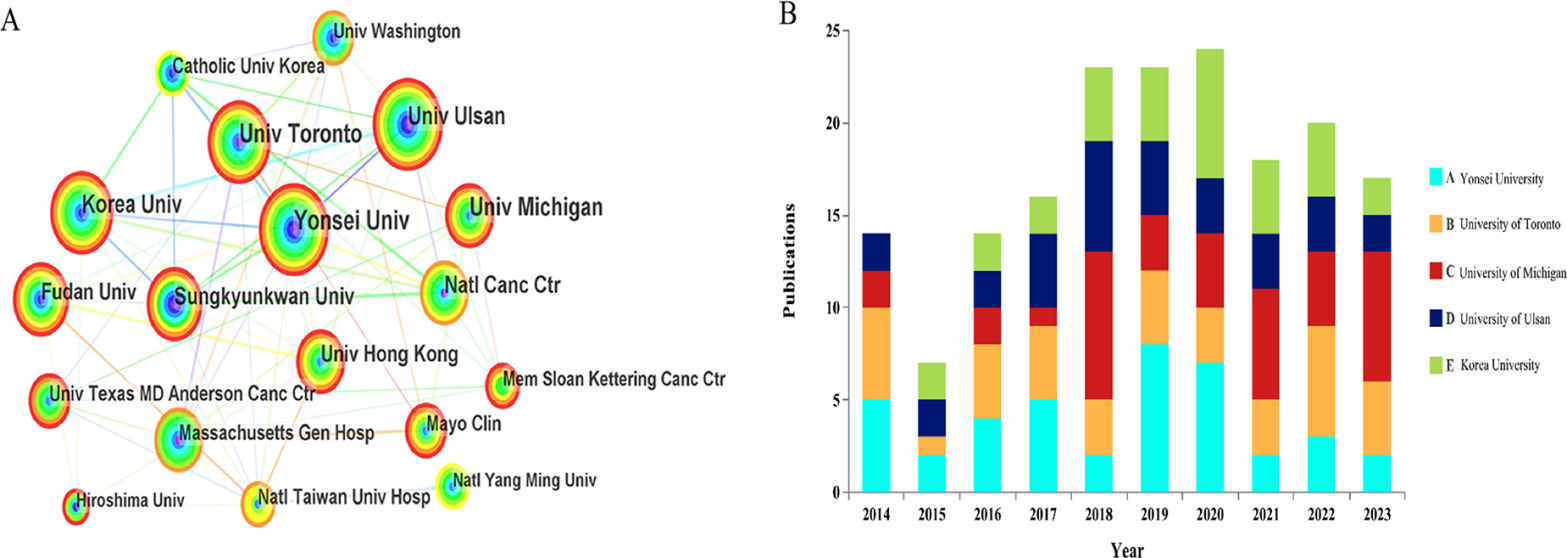
**(A)** The bibliometric analysis of active institutions in the RT and HCC research (the lines represent cooperation relationships, and the colors in the nodes). **(B)** The number of documents published in the last 10 years by the top 5 organizations in terms of publication volume.

**Figure 3A** maps the global distribution of published manuscripts on RT in HCC. Among the 17 participating countries (**Figures 3B, C**), the United States leads in international collaboration, particularly with Canada and China, contributing 147 publications(17.9%), more than any other country, followed by China(n=140), South Korea (n=131), and Japan (n=107). In terms of institutional contributions, 1,083 institutions worldwide have participated in RT studies on HCC. **Figure 4A** identifies the most prolific organizations by publication volume. Of the top 10 publishing institutions, four are from Korea, three from China, and the remainder from the United States, Canada, and Japan(**Table 1**). Yonsei University in South Korea, the University of Michigan in the United States, and the University of Toronto in Canada lead in publication volume, with 40, 37, and 37 documents, respectively, accruing 1,358, 1,777, and 1,263 total citations. The University of Michigan stands out with the highest average number of total citations per publication(n=48). **Figure 4B** presents the publication volume of the top five publishing organizations over the past decade. It highlights that both the University of Michigan and Yonsei University published eight manuscripts in 2018 and 2019, respectively.

**Table 1:** The top 10 institutions contributed to publications in the radiotherapy and HCC research.

Rank	Institution	Country	Counts	TCs	Average TCs
1	Yonsei University	Korea	40	1358	34.0
2	University of Michigan	USA	37	1777	48.0
3	University of Toronto	Canada	37	1263	34.1
4	University of Ulsan	Korea	31	652	21.0
5	Korea University	Korea	31	1033	33.3
6	National Cancer Center	Japan	30	1157	38.6
7	Sungkyunkwan University	Korea	28	776	27.7
8	Fudan University	China	24	466	19.4
9	University of Hongkong	China	23	650	28.3
10	National Taiwan University Hospital	China	22	498	22.6

TCs, total citations.

### Authorship and co-citation analysis

**Figure 5:**
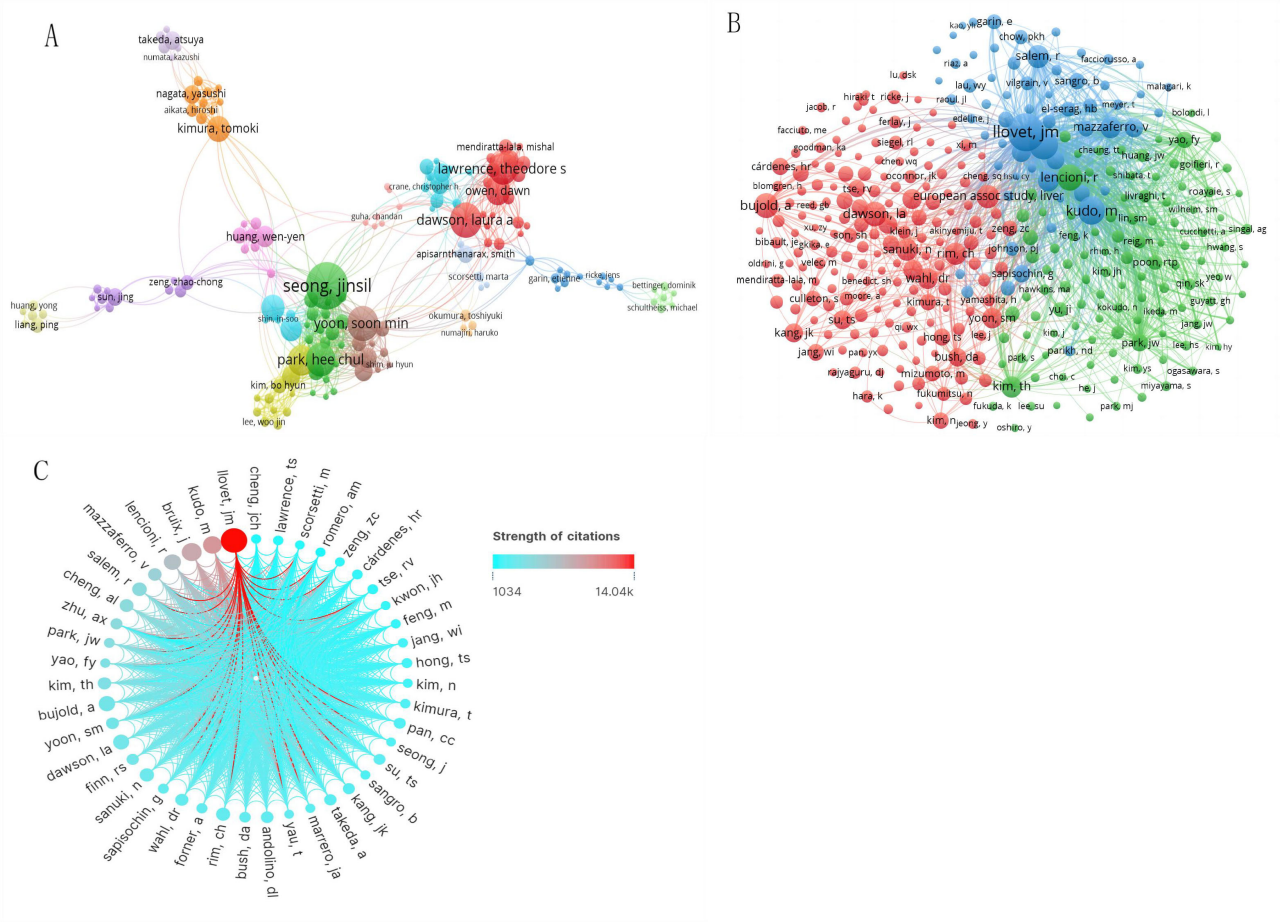
The knowledge map of active and co-cited authors in RT and HCC research. The node size represents the frequency of authors. **(A)** Active authors. **(B)** Active co-cited authors. **(C)** The network map shows the cooperation of prominent co-cited authors (the color of the line shows the strength of citations).

**Figures 5A, B** offer a visualization of the authorship landscape, highlighting key active authors, co-authors, and their collaborative networks. Among more than 4,600 contributors, 44 scholars have published over ten articles, and 13 scientists have attained at least 200 co-citations. Notably, six of the top ten authors are from Korea, aligning with previous institutional and national analyses (**Table 2**). The top authors by publication count are Seong Jinsil, Yoon Soon Min, and Dawson Laura A, with 35, 26, and 26 manuscripts, respectively. As a co-cited author, Llovet JM from the BCLC Group(BCLCG) in Spain has the highest citation count with 776, surpassing his colleague Bruix Jordi(n=440) and Japanese researcher Kudo Masatoshi(n=391). **Figure 5C** illustrates the collaborative networks of prominent co-cited authors, with Llovet JM noted for significant collaborations with researchers such as Kudo Masatoshi and Bruix Jordi.

**Table 2:** The top 10 authors and co-cited authors in the radiotherapy and HCC research.

Rank	Author	Country	Documents	Co-cited author	Country	Institution	TCs
1	Seong Jinsil	Korea	35	Llovet J M	Spain	BCLCG	776
2	Yoon Soon Min	Korea	26	Bruix Jordi	Spain	BCLCG	440
3	Dawson Laura A	Canada	26	Kudo Masatoshi	Japan	Kinki University Hospital	391
4	Park Hee Chul	Korea	25	Lencioni Riccardo	Italy	University of Pisa	327
5	Lawrence Theodore S	USA	24	Salem Riad	USA	Northwestern University	237
6	Kim Tae Hyun	Korea	23	Sanuki Naoko	Japan	Ofuna Chuo Hospital	227
7	Owen Dawn	USA	21	Rim Chai Hong	Korea	Korea University Medical College	220
8	Kimura Tomoki	Japan	19	Cheng Ann-Lii	China	National Taiwan University	213
9	Rim Chai Hong	Korea	19	Mazzaferro Vincenzo	Italy	National Cancer Institute	203
10	Yoon Won Sup	Korea	18	Kim, Tae Hyun	Korea	National Cancer Center	200

BCLCG, Barcelona Clinic Liver Cancer Group.

### Analysis of active journals and co-cited journals

**Figure 6:**
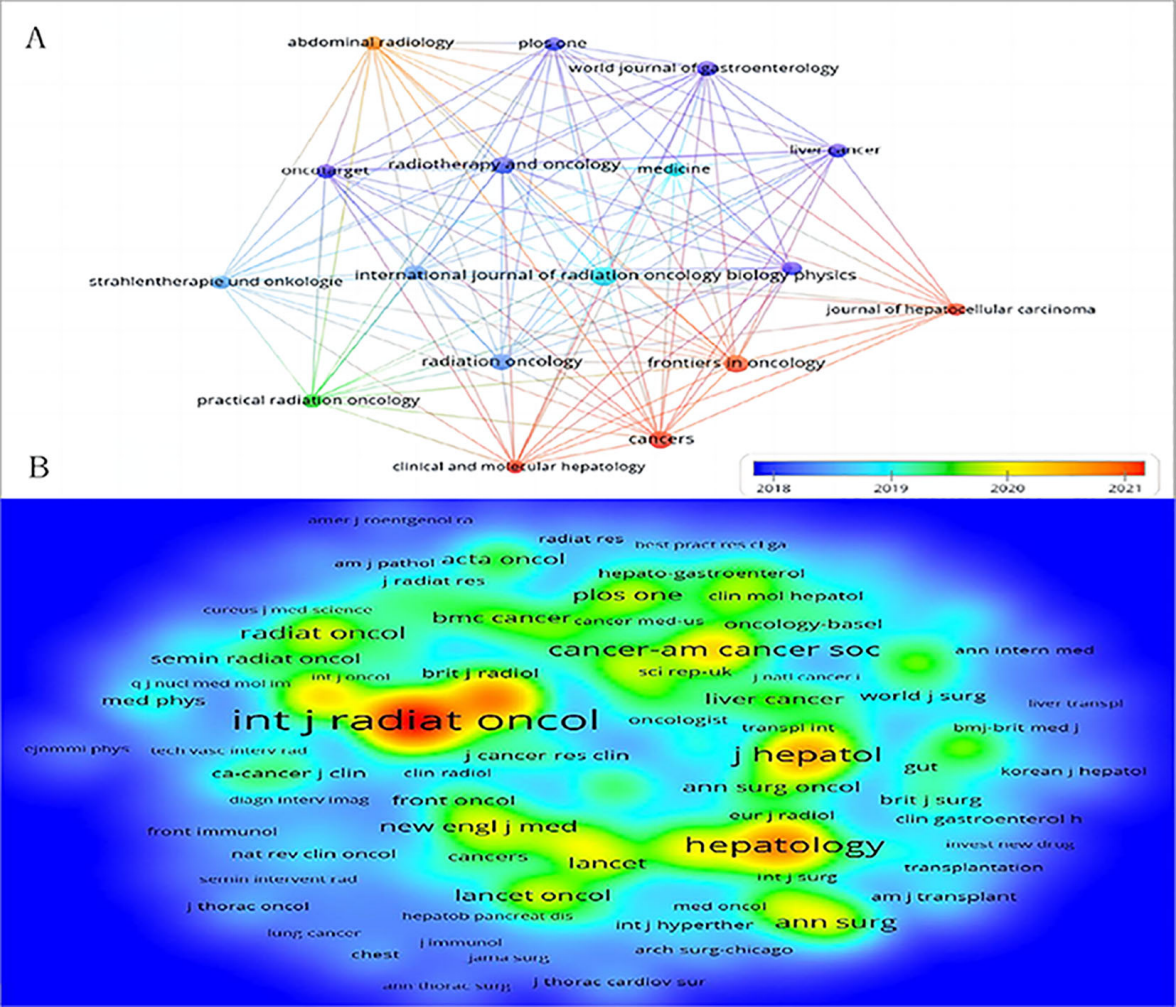
The bibliometric analysis of active journals and co-cited journals in the RT and HCC research. **(A)** Active journals. **(B)** Active co-cited journals. The colors in the nodes represent the years.

From 2014 to 2023, 822 documents on RT and HCC were published across 225 peer-reviewed journals. **Table 3** highlights the top 10 journals based on publication volume. Notably, the *International Journal of Radiation Oncology Biology Physics* leads with 47 publications, and 1,504 total citations. In contrast, the *World Journal of Gastroenterology* achieved the highest average citations per publication (n=42.2), while *Liver Cancer* boasted the highest impact factor(IF). The top 10 journals are all in the Q1 or Q2 quartiles of the Journal Citation Reports(JCR), with 70% belonging to Q1. **Figure 6A** depicts a knowledge map of the most productive journals, highlighting *Journal of Hepatocellular Carcinoma*, *Frontiers in Oncology*, and *Cancers* as emerging journals with rapidly increasing publication volumes.

**Table 3:** The top 10 journals that contributed to publications in the radiotherapy and HCC.

Rank	Journal	Counts	TCs	Average TCs	IF	JCR area
1	International journal of Radiation Oncology Biology Physics	47	1504	32.0	6.4	Q1
2	Frontiers in Oncology	35	304	8.7	3.5	Q2
3	Cancers	32	179	5.6	4.5	Q1
4	Radiotherapy and Oncology	31	1297	41.8	4.9	Q1
5	Radiation Oncology	27	465	17.2	3.3	Q1
6	World journal of Gastroenterology	19	801	42.2	4.3	Q1
7	BMC Cancer	16	318	19.9	3.4	Q2
8	Strahlentherapie Und Onkologie	15	241	16.1	2.7	Q2
9	Liver Cancer	15	447	29.8	11.6	Q1
10	Practical Radiation Oncology	13	206	15.8	3.4	Q1

JCR, journal citation reports; TCs, total citations; IF, impact factor.

Among the 2,282 co-cited journals, 16 had at least 500 citations. **Table 4** lists the top 10 co-cited journals related to RT in HCC. The *International Journal of Radiation Oncology Biology Physics*, published by Elsevier, USA, had the highest number of co-citations (n=3,939). *The Journal of Clinical Oncology*, featuring the American Society of Clinical Oncology, had the highest impact factor (n=42.1). All top 10 co-cited journals are from Q1. **Figure 6B** showcases prestigious co-cited journals such as *The New England Journal of Medicine* (2023IF:96.1, Q1), *The Lancet Oncology* (2023IF:41.6, Q1), and *Gastroenterology* (2023IF:25.7, Q1).

**Table 4:** The top 10 co-cited journals associated with radiotherapy of HCC.

Rank	Journal	TCs	Country	Publisher	IF	JCR area
1	International journal of Radiation Oncology Biology Physics	3929	USA	Elsevier	6.4	Q1
2	Journal of Clinical Oncology	2036	USA	ASCO	42.1	Q1
3	Hepatology	1918	USA	Wiley	12.9	Q1
4	Journal of Hepatology	1622	Netherlands	Elsevier	26.8	Q1
5	Cancer	1113	USA	Wiley	6.2	Q1
6	Radiotherapy and Oncology	1082	UK	BMC	4.9	Q1
7	Radiology	801	USA	RSNA	12.1	Q1
8	Annals of Surgery	731	USA	LWW	7.5	Q1
9	Radiation Oncology	694	UK	BMC	3.3	Q1
10	Gastroenterology	634	USA	Elsevier	25.7	Q1

ASCO, American Society of Clinical Oncology; Wiley, John Wiley & Sons Ltd; BMC, BioMed Central; RSNA, Radiological Society of North America; LWW, Lippincott Williams & Wilkins.

**Figure 7:**
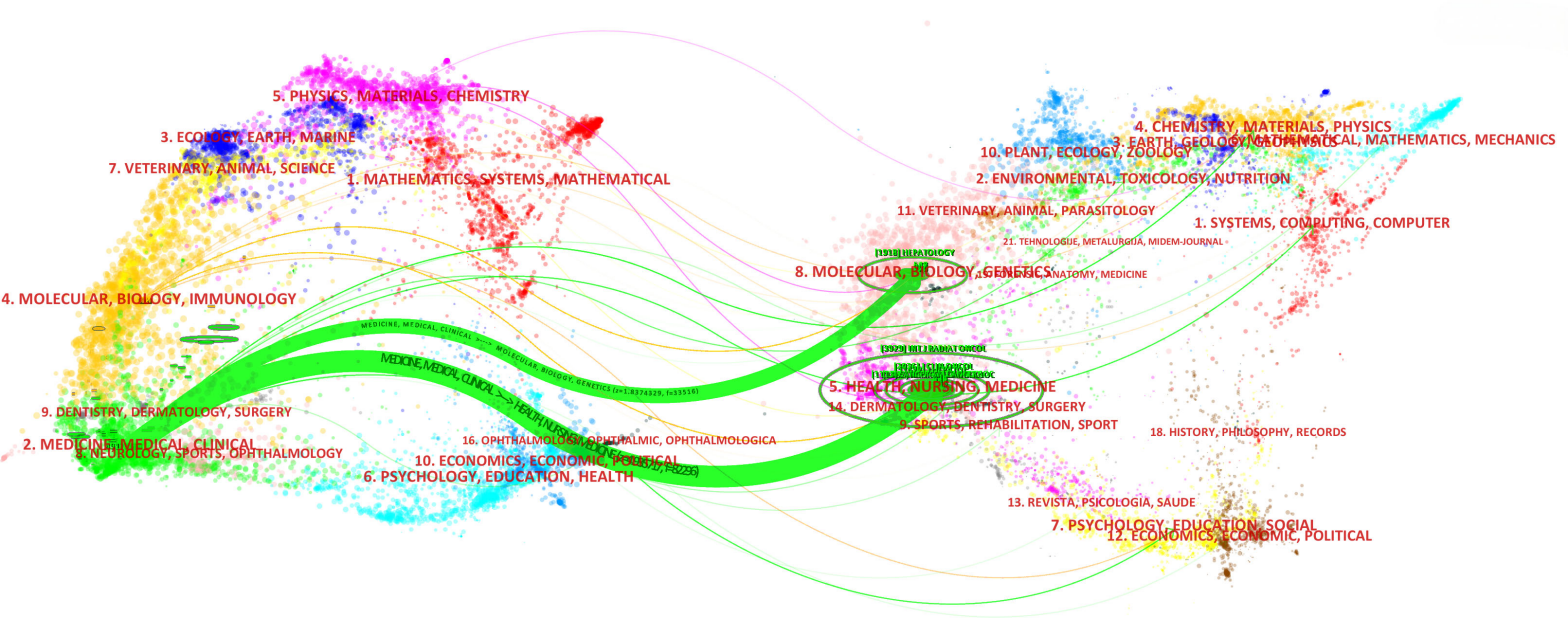
The dual map overlay of journals in the field of RT in HCC (left, citing journals; right, cited journals).

The dual map overlays reflect scientific contributions, illustrating citation networks and validating current research priorities. The Blondel algorithm was employed for journal clustering. The dual map overlay comprises two halves: the left side shows the distribution of journals containing citing documents, and the right side shows journals where cited literature appears. The connecting line represents citation links, with different diameters indicating citation strength. In **Figure 7**, the green main path highlights frequent citations from medicine/medical/clinical journals to those in health/nursing/medicine and molecular/biology/genetics domains.

### Contribution of documents and co-cited references

**Table 5** lists the top 10 most cited documents, comprising seven reviews and three articles. The most cited manuscript, unsurprisingly, is a practice guideline titled “Diagnosis, Staging, and Management of Hepatocellular Carcinoma: 2018 Practice Guidance by the American Association for the Study of Liver Diseases” (30), published in *Hepatology* in 2018, with a substantial 2,855 citations. Additionally, three high-quality articles from *Nature Reviews Gastroenterology & Hepatology* and two from *Nature Reviews Clinical Oncology* are prominent.

**Table 5:** The top 10 cited manuscripts in the field of radiotherapy and HCC.

Rank	Document	Journal	Type	Publication Year	TCs
1	Diagnosis, staging, and management of hepatocellular carcinoma: 2018 practice guidance by the american association for the study of liver diseases	Hepatology	Review	2018	2855
2	A global view of hepatocellular carcinoma: trends, risk, prevention and management	Nature Reviews Gastroenterology & Hepatology	Review	2019	2348
3	Molecular therapies and precision medicine for hepatocellular carcinoma	Nature Reviews Clinical Oncology	Review	2018	1210
4	Hepatocellular carcinoma	Lancet	Review	2022	549
5	Locoregional therapies in the era of molecular and immune treatments for hepatocellular carcinoma	Nature Reviews Gastroenterology & Hepatology	Review	2021	408
6	Outcomes after stereotactic body radiotherapy or radiofrequency ablation for hepatocellular carcinoma	Journal of Clinical Oncology	Article	2016	384
7	Goals and targets for personalized therapy for HCC	Hepatology International	Review	2019	339
8	Personalised versus standard dosimetry approach of selective internal radiation therapy in patients with locally advanced hepatocellular carcinoma (DOSISPHERE-01): a randomised, multicentre, open-label phase 2 trial	Lancet Gastroenterology & Hepatology	Article	2021	296
9	Multi-institutional phase II study of high-dose hypofractionated proton beam therapy in patients with localized, unresectable hepatocellular carcinoma and intrahepatic cholangiocarcinoma	Journal of Clinical Oncology	Article	2016	295
10	2018 Korean liver cancer association-national cancer center korea practice guidelines for the management of hepatocellular carcinoma	Gut and Liver	Review	2019	227

**Figure 8:**
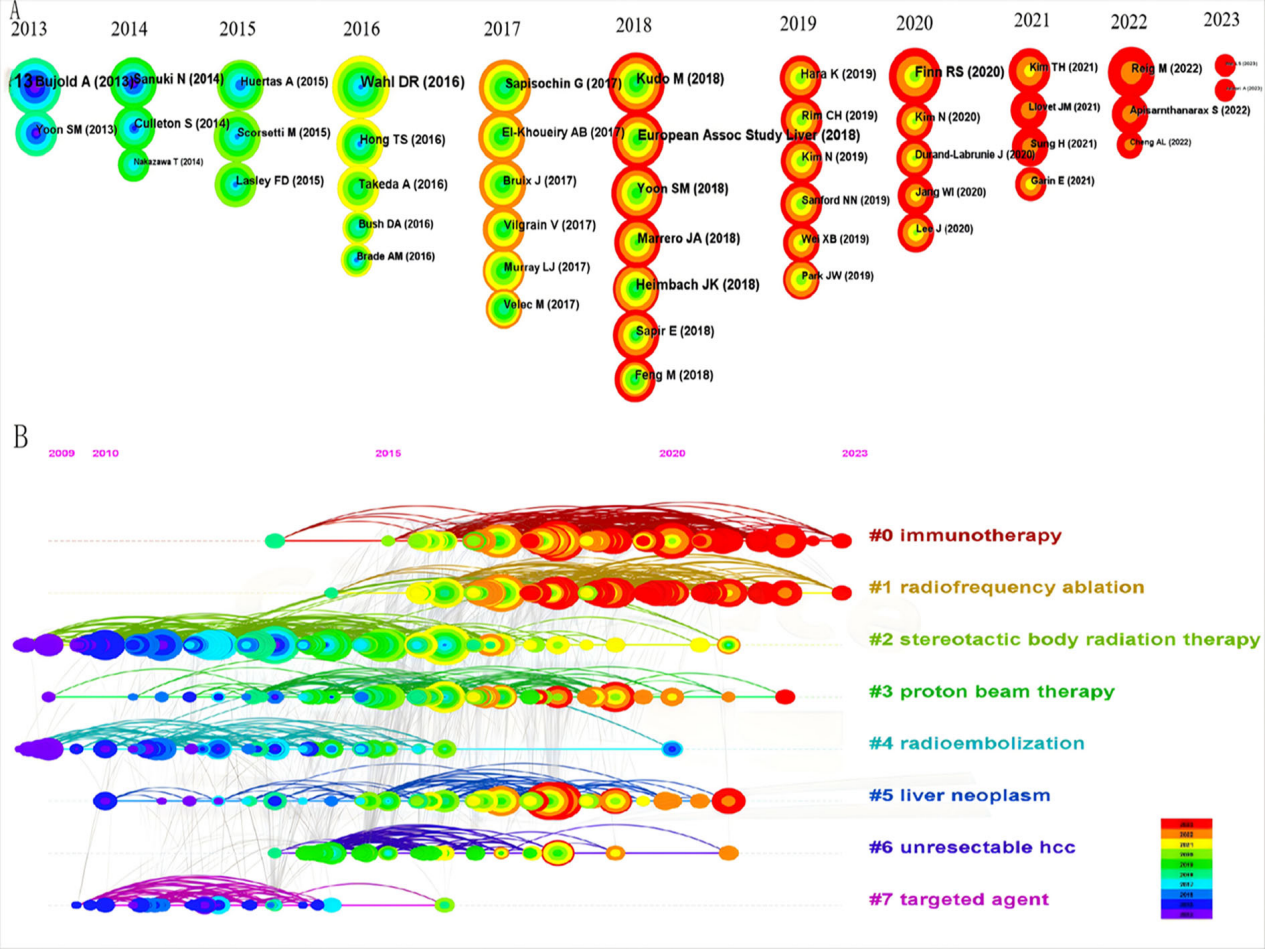
The bibliometric analysis of co-cited references in the RT and HCC research. **(A)** The timezone view. **(B)** The timeline view.

We visualized and analyzed 16,183 co-cited references to highlight the top 10 highly-cited references (**Table 6**), which include eight articles and two reviews. The most co-cited reference is a clinical trial titled “Sequential Phase I and II Trials of Stereotactic Body RT for Locally Advanced Hepatocellular Carcinoma” (31) by Dawson, Laura A., and Bujold Alexis, published in the *Journal of Clinical Oncology* in 2013, with 285 citations. **Figure 8A** uses CiteSpace to analyze co-cited references, creating a timezone map based on publication time and ranking the most cited references annually by circle size from 2014 to 2023. **Figure 8B** presents a timeline view by clustering references, with cluster sizes ranked numerically and smaller values indicating larger clusters. Notably, “0# immunotherapy” is the largest cluster, followed by “1# radiofrequency ablation” and “2# stereotactic body radiation therapy”.

**Table 6:** The top 10 co-cited references of radiotherapy and HCC research.

Rank	Co-cited references	Journal	Type	TCs
1	Sequential phase I and II trials of stereotactic body radiotherapy for locally advanced hepatocellular carcinoma	Journal of Clinical Oncology	Article	285
2	Sorafenib in advanced hepatocellular carcinoma	New England Journal of Medicine	Article	200
3	Outcomes after stereotactic body radiotherapy or radiofrequency ablation for hepatocellular carcinoma	Journal of Clinical Oncology	Article	195
4	Stereotactic body radiotherapy for primary hepatocellular carcinoma	International Journal of Radiation Oncology Biology Physics	Article	174
5	Modified RECIST (mRECIST) assessment for hepatocellular carcinoma	Seminars in Liver Disease	Review	163
6	Radiation-associated liver injury	International Journal of Radiation Oncology Biology Physics	Review	158
7	Stereotactic body radiation therapy for inoperable hepatocellular carcinoma as a local salvage treatment after incomplete transarterial chemoembolization	Cancer	Article	153
8	Efficacy and safety of sorafenib in patients in the Asia-Pacific region with advanced hepatocellular carcinoma: a phase III randomised, double-blind, placebo-controlled trial	Lancet Oncology	Article	141
9	Phase I feasibility trial of stereotactic body radiation therapy for primary hepatocellular carcinoma	Clinical and Translational Radiation Oncology	Article	119
10	Stereotactic body radiotherapy for small hepatocellular carcinoma: a retrospective outcome analysis in 185 patients	Acta Oncologica	Article	118

### Analysis of keywords and burst keywords

**Figure 9:**
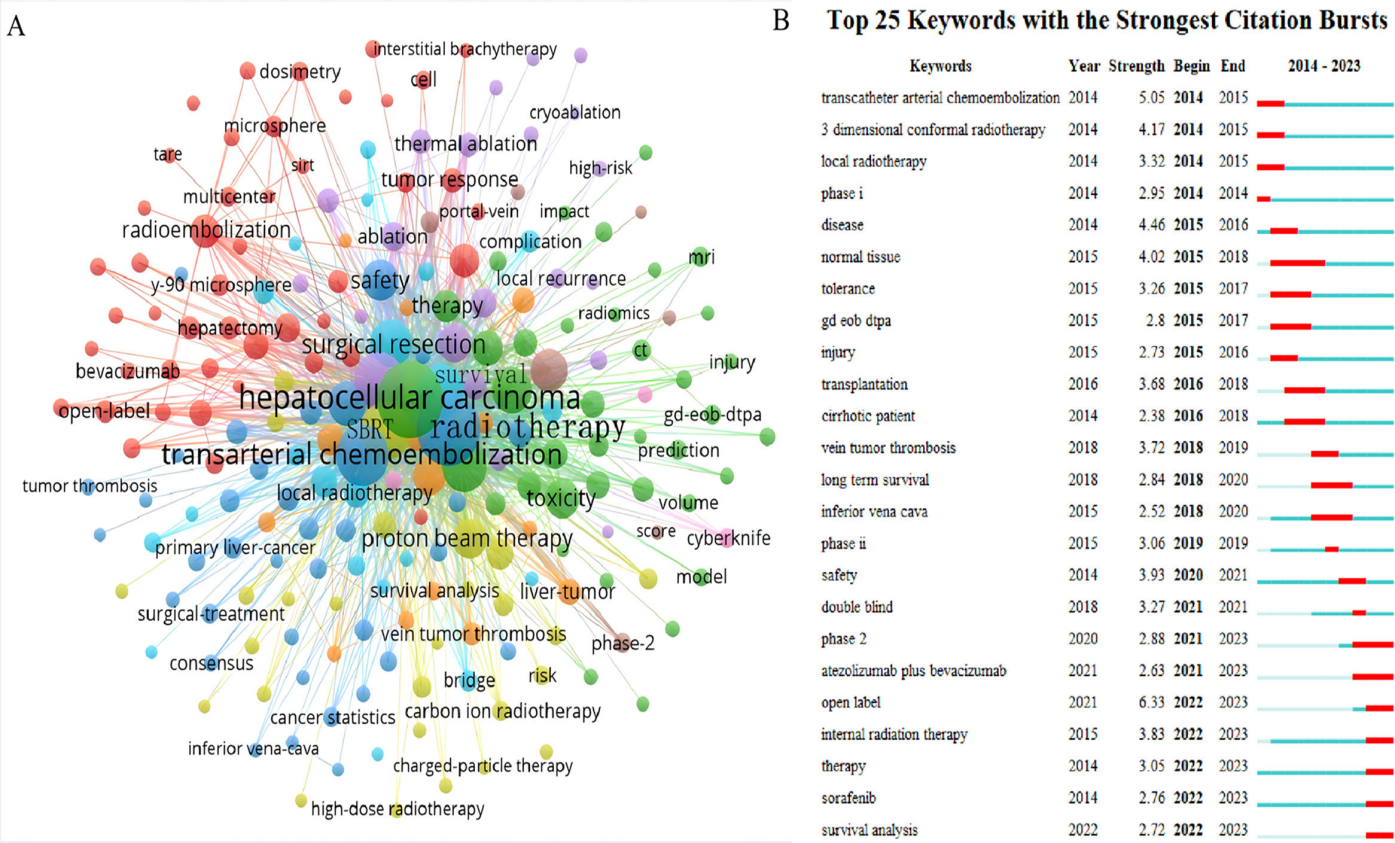
The knowledge map of active keywords and the keywords with the strongest citation bursts in the RT and HCC. **(A)** Active keywords. **(B)** The keywords with the strongest citation bursts.

Keywords encapsulate the core themes of a manuscript, enabling readers to quickly locate relevant literature. This analysis of 822 manuscripts identified 1,887 keywords, with 20 appearing more than 50 times. **Figure 9A** shows that the most common active keywords represent central research topics in RT and HCC. Among these, ‘hepatocellular carcinoma’ was the most frequent keyword, followed closely by ‘radiotherapy’ and ‘transarterial chemoembolization’, each cited over 300 times. ‘Stereotactic body radiation therapy’ and ‘radiofrequency ablation’ have also become prevalent keywords in recent years. A sudden increase in keyword frequency within a specific period often signals an emerging research focus. As shown in **Figure 9B**, ‘atezolizumab plus bevacizumab’, ‘internal radiation therapy’, ‘sorafenib’, and ‘survival analysis’ constituted the most prominent domains from 2021 to 2023.

## Discussion

As a non-invasive local treatment, RT exerts its anti-tumor effects primarily through ionizing radiation, which directly damages biological macromolecules such as nucleic acids, proteins, and enzymes, ultimately causing DNA double-strand breaks (17). Techniques including IMRT, SBRT, and three-dimensional conformal RT(3DCRT) have improved targeting precision and reduced effects on adjacent healthy tissues. The optimal indication for RT in HCC remains somewhat controversial, though consensus supports its use for concurrent PVTT and as a bridging therapy (12–15). Recent advances in the radiotherapeutic management of HCC have led to growing recognition of its indispensable role within multimodal treatment strategies (18–20). This study employs bibliometric methods to quantitatively evaluate the evolution of RT in HCC over the past decade, analyzing publication trends, collaborative networks, and key research themes to enhance comprehension and inform future therapeutic approaches.

### Countries/institutions and their cooperation

The study reveals a substantial expansion in HCC radiotherapy research over the past decade, marked by a pronounced surge from 2017 to 2019 and a peak in 2021. This trend underscores the growing academic engagement with and clinical adoption of RT for HCC. The ten most prolific contributing countries are distributed across Asia (50.3%), the Americas (28.4%), and Europe (21.3%). Although only two developing countries appear on this list, Chinese scientists have contributed 37% of Asia’s publication output, a prominence likely attributable to the higher HCC prevalence in Asia, particularly China, and augmented cancer research funding in certain developing nations (1). Nearly one-third of all publications originate from two countries in the Americas, with the United States alone demonstrating clear leadership in the field. Yonsei University in South Korea is the most productive institution, while only one U.S. institution ranks among the top ten, indicating a geographically uneven distribution of scientific output within the United States. Despite this, the U.S. maintains extensive academic collaborations with more than ten countries, fostering particularly strong partnerships with Canada, European nations such as Germany and Italy, and Asian countries including China and Korea.

### Citation information and impact

High citation counts generally reflect high-quality research that substantially influences innovation and discovery within a field. Studies with more citations typically exert greater scientific impact. This study finds that six of the top ten authors by publication count are from South Korea, which aligns with the country’s overall publication volume. However, despite this high output, only two Korean scholars rank among the top ten co-cited authors, and their influence remains notably lower than that of prominent Spanish scientists Llovet Josep M and Bruix Jordi of the BCLCG. These results suggest that although Korean researchers are prolific, greater emphasis on research quality is needed to achieve broader international recognition. Collaborative efforts can further enhance scientific impact; for example, Llovet Josep M, the most co-cited author, frequently collaborates with Bruix Jordi and Japanese researcher Kudo Masatoshi, who rank as the second and third most co-cited authors, respectively. Their collective contributions have been pivotal in advancing the diagnosis, treatment, staging, and management of HCC (32–35).

High-impact journals typically feature superior research with larger sample sizes, which contributes to their higher impact factors and attracts a broader scholarly audience. The ten most prolific journals all belong to the JCR Q1 or Q2 categories, with Q1 accounting for 70% of these. Notably, these leading journals specialize in oncology rather than covering a broader scope. The International Journal of Radiation Oncology Biology Physics, commonly referred to as the Red Journal, leads in both publication and co-citation counts. As the official journal of the American Society for Radiation Oncology, it focuses on original research involving SBRT combined with TACE or immunotherapy for HCC (36–38). It also highlights technical advances in dosimetry and conformal radiation treatment planning, alongside basic science studies of tumor physiology and the molecular biology of radiation responses in cancer and normal tissues. Radiotherapy and Oncology, known as the Green Journal, ranks fourth in publication volume and sixth in citations. While its impact factor is not exceptionally high, the journal is highly regarded within the field and valued by scientists. It addresses the clinical efficacy of EBRT for HCC with high recurrence risk after hepatectomy and offers evidence supporting proton beam therapy and SBRT for advanced HCC (39–42). Notably, Frontiers in Oncology and Cancers, published by the prolific Swiss publishers Frontiers and MDPI, respectively, have gained prominence over the past three years. Their ascent reflects a growing researcher preference for open-access journals, which enable rapid publication from acceptance to online availability. This trend signals an evolving publishing landscape in which open access and swift dissemination are increasingly prioritized by the scientific community.

Of the ten most cited articles, seven were comprehensive reviews, while the remaining three were clinical trials directly related to radiotherapy (**Table 5**). Six of these ten highly cited publications originated from the United States, with the others coming from Spain, France, and South Korea. This distribution underscores the substantial contribution of American scholars to the field, rather than indicating particularly high productivity from Korean researchers. The most cited article was a review by the AALSD on the integrated management of HCC, which compared various aspects of RT, radiofrequency ablation (RFA), TACE, and SBRT as an alternative to thermal ablation pending prospective randomized studies (30). Significant progress has also been achieved in PBT. In contrast to conventional radiotherapy, which typically employs lower doses with more fractionations, Hong et al. (42) used high-dose, large-fraction PBT for locally unresectable HCC and reported favorable local control (LC) and survival outcomes, including a two-year OS of 63.2%. Although this approach had not been widely validated previously, their study confirms its feasibility and expands the range of available radiotherapy strategies. In a prominent randomized, multicenter, open-label trial, Garin et al. (43) compared individualized and standard dosimetry for selective internal radiotherapy using yttrium-90-loaded glass microspheres in HCC, reporting that individualized dosimetry significantly improved objective response rates(ORR) over standard dosimetry in patients with locally advanced HCC (71% vs 36%). Wahl et al. (44) conducted a retrospective analysis in 2016 comparing the efficacy of SBRT and RFA for unresectable, non-metastatic HCC, addressing a scarcity of data to guide non-surgical management. The RFA group exhibited 1- and 2-year OS rates of 70% and 53%, respectively, while the SBRT group showed OS rates of 74% and 46%. Acute grade 3 or higher complications occurred in 11% of RFA patients and 5% of SBRT patients, a difference that was not statistically significant and indicated comparable safety. One-year and two-year failure-free local progression(FFLP) rates were 83.6% and 80.2% for RFA, compared to 97.4% and 83.8% for SBRT. Furthermore, tumor size significantly influenced FFLP in the RFA cohort but not in the SBRT cohort. For tumors≥2 cm, FFLP was significantly lower with RFA than with SBRT. Collectively, these studies advance the understanding of radiation therapy, offer new therapeutic approaches, and provide compelling, evidence-based support for the radiotherapy treatment of HCC.

### Research hotspots and frontiers

Identifying research hotspots and frontiers is pivotal to understanding the evolution and direction of a scientific field. Key themes and shifts in the research can be illuminated by analyzing the recurring keywords. As shown in **Figures 8**, **9**, these keywords, which appear at prominent points in time, imply the direction of research and guide scientists to constantly push forward the progress of the scientific field.

### Hotspot 1: RT combined with TACE for advanced HCC

This vital hotspot domain includes the following highly crucial clusters, with ‘#2 stereotactic body radiotherapy’ and ‘#6 unresectable HCC’ and several prominent keywords such as ‘transcatheter arterial chemoembolization’, ‘TACE’, ‘stereotactic body radiotherapy’, ‘SBRT’, and ‘survival analysis’.

#### RT combined with TACE in unresectable HCC

TACE has become a cornerstone in the management of HCC, ranking second only to surgical resection and radiotherapy for advanced, unresectable cases (45). As a first-line therapy, TACE yields higher tumor response rates than other non-surgical modalities. Clinical studies confirm that combining TACE with RT remarkably improves ORR (71.8% vs. 53.7%) and enhances 1-, 2-, and 3-year survival relative to TACE alone, while retaining an acceptable safety profile (18). A retrospective cohort study further reported a markedly longer median overall survival in patients with locally advanced HCC receiving TACE-RT combination therapy compared to those treated with sorafenib (14.1 months vs. 3.3 months). Multivariate analysis identified TACE-RT as the sole independent prognostic factor for survival in this cohort (46).

For massive HCC lesions, often exceeding 10 cm in diameter, curative resection is typically not feasible at diagnosis due to vascular invasion or inadequate future liver remnant. Retrospective data indicate that TACE combined with RT significantly prolongs progression-free survival and improves local tumor control in such patients (47). A recent meta-analysis of 25 clinical trials revealed that the TACE-RT group experienced significantly higher 1-year survival rates (HR 0.62, 95% CI 0.51-0.75) and complete response rates compared to the TACE-alone group. This survival advantage progressively increased over 2 to 5 years. Current clinical guidelines therefore recommend TACE-RT combination therapy for unresectable HCC, especially with PVTT (19, 48). However, some recent studies report no prognostic benefit from combining TACE and RT in HCC. A randomized controlled trial(RCT) from Western countries found that although TACE plus RT improved local control, it did not increase progression-free or overall survival and was associated with more frequent liver-related adverse events, primarily hematemesis and ascites (49). This discrepancy may reflect differences in the predominant etiologies of HCC between regions. Alcohol-related liver disease is more common in Western countries, whereas hepatitis B virus(HBV) infection predominates in China. Since therapeutic sensitivity to TACE and RT varies with etiology, these differences may partly explain the lack of efficacy observed with combination therapy in some settings.

#### RT in advanced HCC after incomplete TACE

While TACE remains a cornerstone intervention for HCC, it can inadvertently promote tumor progression by enhancing portal venous blood flow and developing collateral circulation, which may facilitate tumor dissemination while yielding limited apoptotic efficacy. Repeated TACE procedures also risk inducing chemoresistance and iatrogenic hepatocyte damage, ultimately diminishing therapeutic returns. Radiotherapy offers a complementary approach by effectively targeting tumor cells disseminated through collateral circulation, with residual chemotherapeutic agents from prior TACE potentially enhancing radiosensitivity (50). A multicenter phase II prospective trial(N = 121) demonstrated significant clinical benefits of 3D-CRT in patients with unresectable HCC after a suboptimal response to TACE (<3 sessions), achieving 12-week objective response metrics with a 64.5% overall response rate (ORR), comprising 19.4% complete response (CR) and 45.1% partial response (PR) rates (51). In a prospective cohort study (N = 40), early implementation of 3D-CRT following 1–2 unsuccessful TACE cycles produced a 62.8% objective tumor response rate and a 20.9% CR rate, while maintaining acceptable toxicity profiles (52). SBRT delivered at ablative doses (42–60 Gy/3 fractions) after TACE resulted in 6-month CR+PR rates of 38.3%, with subsequent 2-year outcomes of 94.6% LC, 68.7% OS, and 33.8% PFS (53). These collective findings establish RT as a viable salvage therapy following an incomplete TACE response. However, current evidence predominantly originates from single-center retrospective analyses with heterogeneous inclusion criteria, underscoring the need for large-scale prospective trials to validate these observations.

#### SBRT combined with TACE for advanced HCC

International guidelines widely recognize SBRT as a treatment option for HCC across various clinical scenarios (10–15). Most guidelines position SBRT as an alternative or salvage ablation therapy for early-stage HCC and as palliative treatment for symptomatic locally advanced or metastatic disease. TACE similarly receives broad guideline endorsement for unresectable or progressive HCC (10, 11, 14, 15) and represents an integral component of neoadjuvant, conversion, and adjuvant therapeutic strategies (54). When associated toxicities remain manageable, this modality can improve survival outcomes and prognosis (55, 56). Bai et al. (57) retrospectively compared combined TACE-SBRT versus TACE alone for HCC lesions ≤ 5 cm, finding significantly higher 1- and 3-year LC rates (91.1% and 89.9% versus 69.9% and 44.8%; P<0.001) and PFS (56.5% and 32.3% versus 42.2% and 21.6%; P = 0.022) with combination therapy, though OS showed no significant difference(P = 0.206). Multivariate analysis indicated that adding SBRT to TACE did not independently prolong PFS. However, subgroup analysis restricted to patients with fewer than two HCC lesions identified the SBRT-TACE combination as a significant prognostic factor for longer PFS(P = 0.012). The combined approach also failed to improve OS in their cohort significantly. That study (54) suggested two explanations: diverse salvage treatments were administered following recurrence, whether after TACE alone or combination therapy, and baseline liver function was balanced after propensity score matching. We consider this phenomenon may reflect the absence of an overall survival difference between TACE and SBRT recipients after matching (58). Furthermore, Jacob et al. (59) reported that combined TACE-SBRT therapy reduced local recurrence rates compared to TACE alone for HCC lesions≥3 cm (10.8% versus 25.8%, P = 0.04). After censoring for liver transplantation, median survival was significantly longer in the combination group than in the TACE-only group (33 versus 20 months; P = 0.02). These survival improvements occurred without increasing serious adverse event rates. Besides, Kang et al. (60) reported that the thrombus remission rate following treatment by SBRT in patients with HCC and PVTT was remarkably increased. The combination of SBRT and TACE prolongs median survival time and improves the one- and two-year survival rates in patients with HCC complicated by PVTT, compared to SBRT alone.

However, the survival benefit of combined therapy remains debated, with uncertainty over whether it results from the synergy of both modalities or from SBRT alone. Some researchers propose that the favorable outcomes support the complementary roles of TACE and SBRT, where their individual limitations are offset when used together (61). TACE predominantly induces necrosis in the central portion of the HCC tumor, which could reduces tumor volume and may increase the targeted radiation dose while reducing radiation damage to normal liver tissue., whereas SBRT effectively targets peripheral tumor regions. In contrast, several uncontrolled case series have reported tumor control rates as high as 90% with SBRT alone in selected HCC subtypes (62, 63). Moreover, since either TACE or radiotherapy alone is frequently associated with early local recurrence, current clinical practice often combines local modalities in end-stage disease when possible to improve outcomes. Additionally, the sequence and timing interval between TACE and SBRT influence prognosis and adverse reactions during treatment. Existing study (60) suggest that, for patients with PVTT, undergoing SBRT first to reduce tumour volume and improve portal vein blood supply before adding TACE may enhance treatment efficacy and reduce complication rates. Conversely, patients with PVTT who received TACE followed by SBRT demonstrated significantly greater impairment of liver function compared to those treated with SBRT alone. This may be because liver tumour cells exhibit an early response, while normal liver tissue and blood vessels demonstrate a delayed response. These differential responses to radiation therapy may result in early reactions occurring during irradiation or within the first few days or weeks after treatment, while late reactions may emerge months or years later. Consequently, the first two weeks following SBRT may represent a period during which liver tumour cells react to radiation before vascular changes occur. TACE performed during this period may not compromise the therapeutic efficacy of the drugs.

### Hotspot 2: RT in HCC with vascular invasion

The management of HCC with vascular invasion represents a critical area in radiation oncology, characterized by key terms including ‘vascular invasion,’ ‘venous tumor thrombosis,’ ‘tumor thrombus,’ ‘inferior vena cava involvement,’ ‘external beam radiotherapy (EBRT),’ and specifically ‘portal vein tumor thrombosis (PVTT).’ Given the liver’s unique biological and anatomical features, HCC exhibits a strong propensity for invading the intrahepatic vascular system, with PVTT representing its most frequent form. Current evidence reports PVTT incidence rates of 44.0%–62.2% in HCC patients (48, 64–66). This complication markedly worsens prognosis (45), typically accelerating disease progression via intrahepatic dissemination, distant metastasis, and the induction of portal hypertension-related complications like jaundice and ascites. Advanced HCC with PVTT not only exhibits extensive intrahepatic spread but also causes progressive hepatic dysfunction, together contributing to poor clinical outcomes (65, 66). Although conventional therapies such as radical hepatectomy, LT, and TACE face considerable limitations in PVTT management, advances in radiation techniques have established EBRT as an effective and safe option for treating vascular invasion complications, particularly PVTT (19, 67, 68).

#### RT in unresectable HCC with PVTT

For most HCC patients with PVTT, particularly those with type III/IV PVTT under the Oriental Hepatobiliary Surgery Association classification, surgical resection is typically not feasible at diagnosis, which necessitates comprehensive non-surgical management (45). The therapeutic potential of radiotherapy, especially SBRT, in HCC with PVTT stems from its capacity to deliver higher radiation doses to the tumor while minimizing exposure to adjacent normal tissues. This approach may enhance local tumor control and better preserve normal tissue function (66). In a retrospective study of unresectable HCC with vascular invasion, patients received 3D-CRT via 6 or 10 MV X-ray beams, with daily fractions of 1.8–2 Gy to a total dose of 30–56 Gy (69). The ORR was 45%, and responders exhibited a median survival of 13.7 months compared to 5.9 months in non-responders, with liver function class identified as an independent prognostic factor (P = 0.007). Zeng et al. (70) compared outcomes between 44 patients with unresectable HCC and PVTT or IVCTT treated with EBRT and 158 non-EBRT controls. The EBRT group showed superior outcomes, with a median survival of 8 months versus 4 months (P<0.0001) and 1-year survival rates of 34.8% versus 11.4%. Complete thrombus resolution was observed in 34.1% of EBRT-treated patients, while 52.3% exhibited thrombus stability. A propensity-matched analysis of 97 advanced HCC cases further indicated improved median survival with radiotherapy compared to sorafenib monotherapy (5.9 vs. 4.3 months, P = 0.025) (71). Although these studies report encouraging outcomes, most are single-center retrospective analyses with limited sample sizes and preliminary evidence. Multicenter RCTs are therefore needed to validate these findings.

#### RT in resectable HCC with PVTT

Surgical resection remains the most effective treatment for HCC with PVTT when technically feasible (10, 45). PVTT typically originates from the primary tumor site (67, 68, 72), and surgical intervention primarily targets type I/II PVTT under the Oriental Hepatobiliary Surgery Association classification (73). However, surgery is contraindicated for type III/IV PVTT due to poor outcomes (45). RT offers three key clinical benefits in this context: (1) downstaging type III/IV PVTT to enable subsequent resection (74); (2) simultaneous irradiation of primary tumors and thrombi for enhanced LC (66); and (3) survival improvement in advanced HCC with PVTT. Emerging evidence underscores RT’s role in improving prognosis for resectable HCC. A systematic review comparing neoadjuvant RT plus hepatectomy(n=127) versus surgery alone(n=160) across one RCT and two non-RCTs demonstrated superior 1-year OS in the RT cohort (75.2% vs. 43.1%, P<0.001) (67). Non-RCT studies similarly showed prolonged OS with neoadjuvant RT(P<0.0001). Multicenter RCTs confirmed that neoadjuvant RT significantly reduces HCC-related mortality and recurrence compared to surgery alone (75, 76). Furthermore, a single-center RCT reported that postoperative IMRT reduces recurrence rates and prolongs both disease-free survival (DFS) and OS in PVTT cases (77).

### Hotspot3: particle therapy for HCC

This hotspot includes the following clusters and keywords: ‘#1 radiofrequency ablation’, ‘#3 proton beam therapy’, ‘charged particle therapy’, ‘radiofrequency ablation’, ‘RFA’, ‘proton beam therapy’, ‘PBT’, ‘carbon ion radiotherapy’ and ‘CIRT’.

#### Application of PBT in HCC

PBT is an advanced radiotherapy technique that differs from traditional methods by using a beam of protons to deliver targeted radiation to tumor cells, minimizing damage to surrounding healthy tissues (16). PBT is extensively utilized for treating HCC due to its superior deep dose distribution, resulting in excellent LC rates with minimal toxicity (78–81). This is particularly crucial for HCC patients, especially those with large tumors, central locations or impaired liver function (82). Due to their specific anatomical location, tumors in the caudal lobe of the liver represent a challenging target for SBRT and typically have a poorer prognosis. However, a retrospective study involving 30 patients with unresectable caudal lobe HCC treated with PBT reported 1-, 3-, and 5-year LC rates of 100%, 85.9%, and 85.9%, respectively (78). The research also showed 1-, 3-, and 5-year OS rates of 86.6%, 62.8%, and 46.1%, and PFS rates of 65%, 27.5%, and 22%. Meanwhile, a multicenter prospective study in Japan involving 576 cases of unresectable HCC reported a median OS of 48.8 months, median PFS of 14.7 months, a local recurrence rate of just 7.8%, and a 3-year LC rate of 90% during a median follow-up of 39 months (81). Another investigation demonstrated that the 5-year LC rates for BCLC stages 0/A, B, and C were 94%, 87%, and 75%, respectively, showcasing its effectiveness across varying disease stages in 129 HCC cases treated with PBT (83).

Moreover, abundant controlled trials of the efficacy of various treatment modalities for HCC, such as surgical resection(SR), TACE, and RFA, versus PBT have become a hot topic of interest for scientists. Tamura et al. (84) compared SR with PBT for HCC lesions smaller than 10 cm without vessel invasion. While the median survival time favored the SR group(104.1 vs. 64.6 months, P = 0.008), relapse-free survival(RFS) and OS showed no significant difference after propensity-score matching(PSM) (P = 0.099). For untreated HCC eligible for transplantation under Milan or San Francisco criteria, Bush et al. (85) revealed that PBT had similar OS but superior LC rate and PFS compared to TACE, also showing advantages in hospital stay duration and treatment cost. In cases of early-stage HCC where surgery is not feasible, the current guidelines predominantly recommend RFA as the treatment option of choice (11, 12, 45, 86). A retrospective study of 323 new HCC cases demonstrated PBT’s comparable LC (10.4% vs. 7.8%,P=0.895) and acceptable toxicity to RFA (87). In a phase III non-inferiority trial comparing PBT and RFA for recurrent/residual HCC, the 2-year local progression-free survival of PBT was non-inferior to that of RFA, establishing PBT as a safe alternative for small recurrent HCC (size <3 cm, number ≤ 2) (82).

Furthermore, recent advances in proton beam therapy have improved its precision and broadened its clinical applicability, establishing it as a prominent research focus. Respiratory motion significantly influences RT efficacy and the extent of damage to surrounding healthy tissues (88). Respiratory gating stabilizes the density of tissues traversed by the beam, thereby yielding a more uniform dosimetric distribution. Confining the beam-on time to a 40%–50% gating window, as opposed to a 0%-50% ungated window, enhances the consistency of the delivered dose distribution (89). Four-dimensional computed tomography(4D-CT) is now widely employed in radiotherapy; it acquires images over at least one full respiratory cycle and reconstructs them to generate a complete CT dataset representing the entire breathing process (90). The development of four-dimensional cone-beam CT(4D-CBCT) not only mitigates artifacts induced by respiratory motion but also verifies the tumor’s average position, trajectory, and morphological changes during motion before treatment (91). Additionally, evidence indicates that pencil beam scanning(PBS) proton therapy frequently achieves more conformal target dose distributions while reducing irradiation of healthy tissues (92). Wei et al. (93) demonstrated that an advanced pencil beam scanning Bragg peak FLASH-RT technique improves planning outcomes for hepatocellular carcinoma compared to conventional multiple-energy proton PBS delivery.

Collectively, proton beam therapy provides excellent LC and promising survival outcomes for HCC, representing a valuable alternative to traditional therapies, especially in anatomically complex cases or when other modalities entail higher risks. Nevertheless, achieving superior control of respiratory motion during proton beam therapy to increase liver sparing and minimize damage to non-tumor liver tissue warrants further in-depth investigation.

#### Utilization of CIRT in HCC

CIRT has emerged as a promising treatment modality for HCC due to its unique physical and biological properties. Its most notable physical characteristic is the Bragg peak, which enables carbon ions to deliver a concentrated radiation dose to the tumor while sparing surrounding normal tissues (94). The strong biological effectiveness of CIRT stems from reduced lateral scatter and a lower oxygen enhancement ratio, rendering it particularly effective against radioresistant and hypoxic tumors (95–97). Furthermore, the fewer required treatment fractions allow for a shorter overall schedule, improving convenience and compliance for patients with comorbidities or those traveling long distances (94). By preserving liver function and minimizing irradiation of healthy liver regions, CIRT thus expands the options for preventing HCC recurrence (98).

CIRT has also demonstrated excellent outcomes in the clinical management of HCC. The National Institute of Radiological Sciences reported combined results from phase I and II studies, which established maximum tolerated doses of 69.6, 58.0, and 52.8 Gy (RBE) delivered in 12, 8, and 4 fractions, respectively (99). Based on these findings, 52.8 Gy in 4 fractions was selected as the recommended dose for subsequent phase II trials. A Japanese prospective study of 35 HCC patients treated with CIRT (52.8 Gy or 60 Gy in 4 fractions) confirmed 4-year overall survival and local control rates of 69.4% and 76.5%, respectively, with only two patients experiencing grade 3 toxicity and no severe grade 4 or 5 toxicities (100). Meanwhile, a Chinese phase I trial involving 23 HCC patients (median tumor size 4.3 cm) who received CIRT (total dose 55–70 Gy in 5.5-7.0 Gy fractions) reported 5-year OS, PFS, and LC rates of 67.1%, 37%, and 94.4%, respectively, alongside low toxicity, indicating a favorable safety profile (101). In a multi-institutional retrospective study by the Japan Carbon Ion Radiation Oncology Study Group, 174 HCCs were treated with CIRT using regimens of 48 Gy, 52.8 Gy, or 60 Gy, all delivered in four fractions (96). This study reported 3-year local control and overall survival rates of 81.0% and 73.3%, respectively, with only one patient developing grade 4 late dermatitis. The caudate lobe’s deep location within the liver and proximity to major blood vessels, such as the inferior vena cava and portal vein, present technical challenges for surgical resection, RFA, and TACE, often resulting in a poor prognosis for HCC in this region. In such cases, radiotherapy may serve as an effective local treatment option for caudate lobe HCC, as it is less constrained by the lobe’s complex anatomy (102). A small sample study of CIRT for caudate lobe HCC observed no local recurrence and no grade 2 or higher acute adverse events during a median follow-up of 18.3 months (103).

Moreover, most of the available literature focuses only on CIRT, and a few small retrospective studies have examined the effectiveness of CIRT compared with other treatment modalities. Recently, the clinical efficacy of CIRT for HCC has been compared with that of RFA and TACE, and it has been noticed that CIRT has some unique advantages. Fujita et al. (104) retrospectively analyzed HCC cases with CIRT or RFA as initial treatment. CIRT exhibited a significantly lower recurrence rate than RFA in cumulative subsections (2 years: 12.6% vs. 31.7%; 5 years: 15.5% vs. 49.6%). Simultaneously, Shiba et al. (105) demonstrated that CIRT for solitary HCC provided better clinical outcomes than TACE following PSM, with 3-year OS at 88% vs. 58%, PFS at 51% vs. 15%, and LC rate at 80% vs. 26%. While CIRT shows promise based on results from prospective phase I/II and small retrospective studies, the absence of phase III clinical trials directly comparing CIRT with other treatment modalities for HCC is a significant limitation. Future research should focus on conducting large-scale, randomized phase III trials to establish definitive evidence of CIRT’s efficacy and safety compared to other therapies, potentially expanding its role in the treatment of HCC.

However, CIRT for HCC still faces several limitations. As a relatively new and specialized modality, large-scale clinical trials for HCC remain limited. This scarcity of data restricts a comprehensive evaluation of its long-term efficacy and safety. Another major challenge lies in the uncertainty of beam path length (106). The stopping position of carbon ions is sensitive to density variations along their trajectory. Given the steep dose gradients, anatomical variations—such as organ motion or bowel gas—can substantially compromise treatment plan robustness. More robust treatment planning and motion management strategies are therefore required to mitigate these uncertainties. Moreover, the relative biological effectiveness of CIRT itself is also subject to uncertainty (107).Currently, the three major RBE calculation models employed in global carbon ion therapy are the mixed-beam model, the local effect model (LEM), and the microdosimetric kinetic model(MKM), each of which possesses its own distinct physical and clinical application characteristics. In the mixed-beam model, the RBE is calculated based on photon and ion cell survival curves using specified survival levels (108). The dose distribution curve and its central value for spread-out Bragg peak(SOBP) must be determined at the survival level corresponding to the absorbed dose. However, since the RBE curve for each SOBP is encoded within its corresponding edge filter, specific hardware components are required for each dose level (94). Given the clinical impracticality of this approach, the mixed-beam model disregards RBE dose dependency, setting all RBE values relative to the 10% survival threshold-even for highly segmented treatments (109). Within the framework of the mixed-beam model, the clinical dose merely indicates treatment efficacy. The optimal dose and fractionation regimen must be established via clinical trials (110). The LEM explains increased stochastic radiation effects by exploiting the microscopic characteristics of energy deposition around ion trajectories,which is widely used in European countries (111). Unlike Japan’s mixed-beam model approach, LEM’s clinical application considers late effects in normal tissues to be clinically relevant endpoints (109). As LEM is combined with active beam scanning technology, the RBE distribution is not tied to any patient-specific hardware. Consequently, the dose dependency of the RBE can be easily evaluated by adjusting the delivered absorbed dose distribution (109). Furthermore, providing multiple fields per fraction is feasible because LEM calculates the RBE of the local particle spectrum.The MKM is derived from microdosimetric biological models and is based on the theory of dual radiation action (112). With the introduction of more flexible beam scanning techniques in the National Institute of Radiological Sciences in 2011, the MKM was clinically implemented and subsequently replaced the phenomenological mixed beam model in passive beam lines (113). The purpose of introducing the MKM was to account for the dose-dependent nature of RBE and its dependence on the fragment spectrum generated by primary ions. This approach is also regarded as a pragmatic strategy designed to maintain consistency with previous dose prescriptions employing hybrid beam models, thereby enabling identical clinical outcomes under the same prescribed doses (113). Neither of these dependencies was incorporated in the hybrid beam model. Consequently, the MKM can also be applied to multi-field optimization in IMPT (113). RBE is affected by numerous factors, including measured endpoint, dose, dose rate, dose per fractionation, number of fractions, and cell cycle phase. Theoretical modeling of the biological effects of heavy ions remains a challenging task due to the complexity and limited knowledge of the physical, chemical, and biological processes involved.

### Hotspot4: systemic therapy combined with RT for advanced HCC

The presence of burst keywords implies domains of intense research activity and potentially groundbreaking advancements. This pivotal hotspot area includes the following highly crucial clusters, with ‘0# immunotherapy’ as the top cluster of all clusters and ‘7# targeted therapies’ and some prominent burst keywords such as ‘sorafenib’ and ‘atezolizumab and bevacizumab’. The phenomenon illustrates a vital trend and growing interest in the roles of targeting and immunotherapy combined with RT in advanced HCC.

#### Combination of targeted therapy and RT for advanced HCC

Current therapeutic strategies for advanced HCC increasingly emphasize the potential of combination approaches, particularly integrating RT with systemic agents such as targeted therapies and immunotherapies in recent years. Sorafenib, the first tyrosine kinase inhibitor(TKI) approved for HCC based on the landmark SHARP trial, represents a pivotal milestone in advanced HCC management (114). However, acquired resistance to sorafenib, observed in approximately 70% of patients, continues to pose a major clinical challenge (115). Emerging clinical data suggest synergistic effects when combining RT with targeted strategies. A Korean retrospective cohort study demonstrated that sequential administration of local RT following sorafenib significantly improved OS compared to sorafenib monotherapy in BCLC stage C HCC, particularly among patients receiving sorafenib for>12 weeks (20). Similarly, a meta-analysis of 11 studies revealed that concurrent sorafenib and EBRT provided superior OS benefits compared to sequential administration (116). Lenvatinib is another option for first line therapy and is preferred because of better tolerability and longer time to tumour progression compared to sorafenib (117).A PSM reported SBRT plus lenvatinib is expected to significantly improve OS, PFS, and ORR for advanced HCC when compared to SBRT alone, with manageable adverse effects (118).

Notably, the combination strategy shows particular promise in HCC with PVTT. Prospective studies suggest that combining IMRT with sorafenib in HCC patients with PVTT may enhance long-term tumor control without significantly increasing treatment-related toxicity (119, 120). Similarly, the combination of lenvatinib and liver-directed RT is relatively safe and effective in increasing the intrahepatic tumor response and improving PFS in HCC and macroscopic tumor thrombosis (121). Moreover, due to the poor prognosis of type IV PVTT, most phase III trials exclude this population. Li et al. (122)stated that lenvatinib in combination with immunosuppressants and radiotherapy has shown promising results, with OS and PFS of 9.4 and 4.9 months, respectively, and an ORR of 61.5% in mRECIST-based PVTT, with no treatment-related deaths. Simultaneously, a multicentre trial reported that bevacizumab combined with RT and sinidlizumab in the treatment of PVTT achieved an ORR of 58.7% and a 100% disease control rate without unexpected adverse events or treatment-related deaths (123). Then, a Chinese multicenter cohort study demonstrated that combined modality therapy significantly reduced the risk of pulmonary metastasis progression and improved survival outcomes compared to RT alone (124). Nevertheless, a phase II trial reported no statistically significant OS difference between conventional EBRT combined with sorafenib versus EBRT alone in BCLC stage B/C HCC (median OS: 9.9 vs. 9.6 months; P = 0.544) (125). These divergent findings highlight the need for rigorous patient selection criteria and standardized treatment protocols in future RCTs.

Notably, the toxic reactions observed in trials combining targeted agents with radiotherapy warrant attention. These toxicities primarily involve hepatotoxicity and gastrointestinal toxicity. In a phase 2 study of sorafenib combined with radiotherapy for hepatocellular carcinoma, investigators highlighted hepatotoxicity as a key safety concern for this combination therapy (126). Multiple trials have reported hepatotoxicity incidences of 0–19% in the liver SBRT cohort and 3–18% in the conventionally fractionated liver radiotherapy cohort, rates that appear elevated compared to sorafenib alone (127, 128). These findings suggest a volume-dependent effect when liver radiotherapy is combined with sorafenib, indicating that radiotherapy enhances TKI-associated hepatotoxicity. The most serious gastrointestinal toxicities include bleeding and perforation, with acute-phase incidence rates reaching 19% (129); since previous sorafenib monotherapy trials did not report such severe gastrointestinal events, this increase is reasonably attributed to radiotherapy. Furthermore, available evidence indicates that sorafenib may exert a radiosensitizing effect, potentially elevating the risk of gastrointestinal perforation (130). The dose–volume tolerance of the liver is well established, and multivariate analyses confirm that mean liver dose predicts radiation-induced liver disease (131), helping explain the elevated toxicity under combination therapy, as VEGF pathway inhibition likely impedes recovery from radiation-induced vascular injury. This synergy between VEGF inhibition and radiotherapy-induced microvascular damage in the bowel increases the risk of severe gastrointestinal toxicity during combined anti-angiogenic and radiotherapy treatment (129). However, most supporting data originate from early radiotherapy trials in hepatocellular carcinoma; with advances in radiotherapy technology and improved understanding of radiation-induced liver disease, recent studies generally indicate that both hepatotoxicity and gastrointestinal toxicity remain manageable.

#### Integration of immunotherapy and RT for advanced HCC

Immune checkpoint inhibitors (ICIs) block co-stimulatory and inhibitory signals that govern T-cell activation, thereby overcoming immune resistance mechanisms (132). Agents such as atilizumab and nivolumab enhance systemic antitumor immunity by targeting immunomodulators, including programmed cell death ligand 1(PD-L1) or programmed cell death 1(PD-1) (129). One study further revealed that radiotherapy induces PD-L1 expression in tumor cells and supports the antitumor potential of anti-PD-L1 agents in HCC (117). Radiotherapy and immunotherapy appear to interact by augmenting antigen delivery to the immune system and stimulating local immune activity within irradiated tissue, which can occasionally produce a durable antitumor response associated with sustained tumor regression (129, 132, 133). Consequently, combining RT with ICIs is expected to produce synergistic effects. A Chinese retrospective analysis reported that incorporating high-dose EBRT into atezolizumab and bevacizumab therapy improved objective response rate (50.0% vs. 11.8%) and overall survival (not reached vs. 5.5 months) in patients with highly advanced HCC presenting with portal vein tumor thrombosis or tumors occupying over 50% of liver volume, while maintaining a comparable incidence of severe (grade≥3) adverse events (14.3% vs. 14.7%) (134). Similarly, a phase II trial revealed that concurrent EBRT with nivolumab significantly prolonged progression-free survival, overall survival, and objective response rate in HCC patients with macrovascular invasion, without compromising safety (135).

Clinical trials combining RT with ICIs have not revealed adverse effects comparable to those observed with tyrosine kinase inhibitors and RT. Studies indicate that ICI-RT combinations are well tolerated, with safety profiles consistent with monotherapy and no grade 5 toxicities reported. The most frequent grade 3–4 toxicities potentially related to radiotherapy included elevated liver enzymes (0-14%), colitis (0-11%), and pneumonia (0-10%) (129). Outside the radiation field, serious toxicities were predominantly gastrointestinal or hematological. Importantly, ICIs can induce delayed immune-related adverse events (136); although none of the included trials reported such events, their occurrence may become evident as more patients receive combination treatment.

Despite these promising early results, large-scale, multicenter, prospective trials specifically examining systemic therapy and RT combinations in HCC remain scarce, particularly regarding radiation dosing, fractionation schedules, and sequencing with sorafenib or ICIs. Further investigation is necessary to define the optimal integration of these treatment modalities, which may ultimately improve outcomes for advanced HCC. Research into combination strategies, especially those incorporating radiotherapy and systemic agents such as sorafenib or ICIs, could reveal new opportunities to enhance therapeutic efficacy and survival in this challenging disease context.

## Research limitations

This study systematically evaluated the development of RT in HCC over the past 10 years, acknowledging the key contributions made to the evolution and advancement of this specialized field. Indeed, certain limitations have also been noted in this study. The data source is only based on the WoSCC, albeit a very professional and scientific one, which can’t fully cover relevant publications. Moreover, only articles and reviews written in English were retrieved in our analysis, which might result in incomplete data collection from other types of publications. This research only analyses information on basic publications and lacks a detailed examination of their specific content. Additionally, as citations include self-citations, which we have not included in this study, this may contain controversial literature. Although these biases of this study, our results are still a true and reliable reflection of the research evolution of RT in HCC over the past 10 years.

## Conclusions

This study comprehensively examines research trends and quantitatively analyzes the key areas and emerging frontiers of RT in HCC using a visual analysis strategy. Over the past decade, there has been a steady increase in the number of publications on RT and HCC. Notably, there is significant collaboration among researchers and institutions in the United States, Canada, and China, with the United States emerging as a leading nation in RT research for HCC. The utilization of RT for HCC with PVTT and the integration of TACE in the comprehensive treatment of advanced HCC have received substantial attention. Moreover, recent studies have highlighted the particle therapy and combination targeted immunotherapies with RT, in particular, how to better control respiratory gating techniques during PBT treatment and more robust treatment planning and motion management techniques in CIRT. Then, the dosing, sequencing, and intervals of targeted immunotherapies in conjunction with RT, as well as clinical adverse effects in multicentre trials, are essential topics and emerging areas of interest. These findings offer a detailed foundation for clinicians and researchers, providing valuable insights that facilitate subsequent studies and help streamline the process of identifying specific topics and fields for investigation.

## Data Availability

The datasets presented in this study can be found in online repositories. The names of the repository/repositories and accession number(s) can be found in the article/**Supplementary Material**.
