# Supplementary material for: The research perspectives and frontiers on radiotherapy for hepatocellular carcinoma: a bibliometric analysis and systematic review
Source: Front Oncol. 2025 Dec 16;15:1601207. doi: 10.3389/fonc.2025.1601207 (PMC12710505; doi:10.3389/fonc.2025.1601207)
Supplement: Supplementary file 1 [file Table1.docx]

TS=( (primary hepatic cancer) OR (primary liver cancer) OR (hepatocellular carcinoma) OR (malignant hepatoma) OR (primary liver carcinoma)) OR (primary hepatic carcinoma) OR (HCC) ) AND ((stereotactic body radiation therapy) OR (image guided radiation therapy) OR (proton beam radiation therapy) OR (stereotactic ablative radiation therapy) OR (external beam radiation therapy) OR (intensity-modulated radiation therapy) OR (three-dimensional conformal radiation therapy) OR (targeted radiation therapy) OR (radiotherapy) OR (SBRT) OR (IMRT) OR (PBT) )
