# Supplementary material for: The research perspectives and frontiers on radiotherapy for hepatocellular carcinoma: a bibliometric analysis and systematic review
Source: Front Oncol. 2025 Dec 16;15:1601207. doi: 10.3389/fonc.2025.1601207 (PMC12710505; doi:10.3389/fonc.2025.1601207)
Supplement: Supplementary file 2 [file Table2.docx]

Professional term abbreviations

| Term | Abbreviation |
| --- | --- |
| Radiotherapy | RT |
| Hepatocellular carcinoma | HCC |
| Science Citation Index-Expanded | SCIE |
| Web of Science Core Collection | WoSCC |
| Transarterial chemoembolization | TACE |
| Portal vein tumor thrombosis | PVTT |
| Primary liver cancer | PLC |
| Liver transplantation | LT |
| Radiation-induced liver disease | RILD |
| Stereotactic body radiotherapy | SBRT |
| Intensity-modulated radiotherapy | IMRT |
| Image-guided radiotherapy | IGRT |
| American Association for the Study of Liver Diseases | AASLD |
| Barcelona Clinic Liver Cancer | BCLC |
| National Comprehensive Cancer Network | NCCN |
| European Association for the Study of the Liver | EASL |
| Selective internal radiotherapy | SIRT |
| European Society for Medical Oncology | ESMO |
| Proton beam therapy | PBT |
| Carbon ion radiotherapy | CIRT |
| Survival rates | OS |
| Consolidated Criteria for Reporting Qualitative Research | COREQ |
| Three-dimensional conformal RT | 3DRT |
| Radiofrequency ablation | RFA |
| Randomized controlled trial | RCT |
| Complete response | CR |
| Partial response | PR |
| Objective response rate | ORR |
| Inferior vena cava tumor thrombosis | ICVTT |
| Disease-free survival | DFS |
| Local control | LC |
| Surgical resection | SR |
| Relapse-free survival | RFS |
| Propensity-score matching | PSM |
| Four-dimensional CT | 4D-CT |
| Four-dimensional cone beam CT | 4D-CBCT |
| Pencil beam scanning | PBS |
| Tyrosine kinase inhibitor | TKI |
| Vascular endothelial growth | VEGF |
| Immune checkpoint inhibitors | ICIs |
| Programmed cell death ligand 1 | PD-L1 |
| Programmed cell death 1 | PD-1 |
